# Evaluating the effects of two different kinesiology taping techniques on shoulder pain and function in patients with hypermobile Ehlers-Danlos syndrome

**DOI:** 10.3389/fpain.2023.1089748

**Published:** 2023-01-16

**Authors:** Frank Tudini, David Levine, Michael Healy, Max Jordon, Kevin Chui

**Affiliations:** ^1^ Department of Physical Therapy, The University of Tennessee at Chattanooga, Chattanooga, TN, United States; ^2^ Healy Physical Therapy and Sports Medicine, East Providence, RI, United States; ^3^ Department of Physical Therapy, Radford University, Roanoke, VA, United States

**Keywords:** physical therapy, shoulder pain, kinesiology tape, functional outcome measures, Ehlers-Danlos syndrome-hypermobility type

## Abstract

**Background:**

Ehlers-Danlos Syndrome (EDS) is a group of inherited connective tissue disorders which predominantly affects women and has a prevalence as high as 1 in 5,000 individuals. Hypermobile EDS (hEDS) is the most common subtype of EDS and is characterized by multi-joint pain, particularly in large joints such as the shoulder. Physical therapy is often utilized to address the pain, physical impairments, and functional loss in patients with EDS. Kinesiology Tape (KT) is an intervention commonly used by physical therapists for treating shoulder pain and dysfunction. Studies related to the effectiveness of KT in patients with shoulder pain is equivocal and there are a lack of studies specifically studying the effects of KT in an EDS population.

**Purpose:**

The purpose of this study was to assess the efficacy and short-term effects of two different KT techniques on shoulder pain and function in individuals with hEDS and shoulder pain.

**Methods:**

Participants were recruited from EDS support groups in the New England area of the United States; were diagnosed with hEDS by their physician; and had shoulder pain. Baseline demographic information was obtained for each participant followed by completion of 4 patient reported outcome (PRO) measures: the Upper Extremity Functional Index, QuickDASH (Disabilities of the Arm, Shoulder, & Hand), Shoulder Pain and Disability Index, and the Western Ontario Shoulder Instability Index. Current pain level, average pain over the past 24 h, and worst pain over the past 24 h were recorded using the numeric pain rating scale (NPRS). Subjects were randomly assigned to receive either an experimental shoulder KT procedure or a control shoulder taping. Immediately after taping, the NPRS was reassessed. Subjects then returned 48 h later to repeat the NPRS and PRO measures.

**Results:**

There was no significant difference between the experimental and control tape groups for any outcome measure. There was a significant improvement from pre-taping to 48-hours post taping for each of the 4 PRO measures with large effect sizes (*p* < 0.001; *ƞ*_p_^2^ = .517–.719). Likewise, average, and worst pain over the last 24 h significantly improved with large effect sizes over the same period (*p* = 0.005; *ƞ*_p_^2^ = .225 and *p* < 0.001; *ƞ*_p_^2^ = .382, respectively). Current NPRS levels significantly improved from pre-tape to immediately post-tape (*p* = .023, *ƞ*_p_^2^ = .131) and was maintained through the 48-hour follow up, although no further improvement was seen.

**Conclusion:**

KT is an inexpensive and relatively safe intervention that is easy to apply and can offer temporary improvements in pain and function for patients with EDS and shoulder pain.

## Introduction

Ehlers-Danlos Syndrome (EDS) represents a group of inherited connective tissue disorders which predominantly affects women and has been traditionally thought to affect approximately 1 in 5,000 individuals (1). More recent research has shown that the prevalence may in fact be much higher. In Wales, 0.19% have a combined diagnosis of EDS and joint hypermobility syndrome (2) and other research has proposed a prevalence of symptomatic general joint hypermobility as high as 0.75%–2% of the population (3). While there are 13 subtypes of EDS, the hypermobile type (hEDS) is the most prevalent, likely representing 80%–90% of the cases (4). Hypermobile EDS is a clinical diagnosis assigned when individuals meet the following 3 criteria. The first criterion is generalized joint hypermobility, defined as a Beighton score of ≥6 for pre-pubertal children and adolescents, ≥5 for pubertal men and women up to the age of 50 years, and ≥4 for those >50 years of age (5). The second criterion involves the exclusion of other types of EDS, connective tissue disorders, and alternative diagnoses. The third criterion includes 2 of the following 3 items: the presentation of systemic manifestations of more generalized connective tissue disorder (skin hyperextensibility, unexplained striae, or recurrent abdominal hernias for example), a positive family history, and/or musculoskeletal complications (e.g., chronic pain, recurrent dislocations etc.) (5).

Musculoskeletal pain affecting more than 1 joint is a common manifestation of hEDS, with some studies reporting its presence in 100% of subjects (6, 7). The joint pain most commonly affects larger joints with greater ranges of motion, such as the shoulder, where recurrent subluxations often occur (6, 8). In one study, 78% of patients with hEDS demonstrated some type of shoulder pathology (6). In addition to pain, patients with hEDS demonstrate reduced muscle strength, endurance, and proprioception (7, 9). As pain becomes chronic and joint instability progresses, activities of daily living become increasingly affected. Physical therapy (PT) is often utilized to address the above listed impairments and functional limitations and is considered a primary treatment for hEDS (5, 10). PT interventions including low-impact exercises for muscle strengthening and joint stabilization, proprioceptive training, and patient education (10). Another intervention commonly used in patients with shoulder pain is kinesiology tape (KT).

It is theorized that when KT is applied directly to the skin to treat musculoskeletal injuries, it stimulates the afferent nerves and mechanoreceptors of the skin, joints, and soft tissues to enhance proprioception (11–13). The effectiveness of this intervention is equivocal. In individuals with shoulder dysfunction, KT has been shown to improve scapular joint position sense and movement control (14). Other studies report decreases in pain and subluxation in patients with hemiplegic shoulder pain after stroke (15, 16). In contrast, other studies have shown that KT tape was not effective for improving pain and function in patients with shoulder pain (17, 18). There is a lack of research examining the effectiveness of KT in a population with EDS. The purpose of this study, therefore, was to assess the efficacy and short-term effects of two different KT techniques on shoulder pain and function in individuals with hEDS and shoulder pain. It was hypothesized that KT would decrease pain and improve function.

## Materials and methods

Participants in this study were recruited from EDS support groups in the New England area of the United States and were all diagnosed with hEDS by their medical physician. The study was performed in a PT clinic in Rhode Island, USA that specializes in the treatment of individuals with EDS. Inclusion criteria included a diagnosis of hEDS, unilateral or bilateral shoulder pain, a positive shoulder apprehension test, and a Beighton score of≥5/9. Exclusion criteria included past shoulder surgery, cervical surgery, cervical injury within the last 12 months, and/or pregnancy. This study was approved by the University of Tennessee at Chattanooga Institutional Review Board (#20-040) and informed consent was obtained from all subjects.

Prior to arrival, the names of the participants were placed in a bin, randomly drawn, and alternately placed in the experimental or control taping group. The participants were blinded to the group placement. Upon arrival and after gaining informed consent, baseline demographic information was obtained, the shoulder apprehension test was performed, and a Beighton score was calculated. Each participant completed 4 patient reported outcome (PRO) measures including the Upper Extremity Functional Index (UEFI), QuickDASH (Disabilities of the Arm, Shoulder, & Hand), Shoulder Pain and Disability Index (SPADI), and the Western Ontario Shoulder Instability Index (WOSI). Current pain level, average pain over the past 24 h, and worst pain over the past 24 h was recorded using the numeric pain rating scale (NPRS). As all subjects reported bilateral shoulder pain, they were asked to score the PRO measures and NPRS using the upper extremity that had the highest average pain.

The UEFI is a standardized, reliable and validated measure that is used to assess upper extremity functional impairments in individuals with musculoskeletal upper limb dysfunction (19, 20). The minimally clinically important difference (MCID) was found to be 8.0/80 (19). The QuickDASH contains a subset of 11 items taken from the 30-item Disabilities of the Arm, Shoulder & Hand (DASH) outcome measure (21). The QuickDASH is a validated measure (22) with excellent reliability (23). The MCID for individuals with shoulder pain is 8.0/100 (22). The SPADI is a validated measure that has been shown to be reliable in patients with shoulder dysfunction (24). The MCID was found to be 13.2/100 in subjects with upper extremity dysfunction (25). The WOSI is a commonly used PRO measure for patients with shoulder instability (26, 27). The total score of the WOSI ranges from 0 to 2100, with 0 indicating no limitations and 2,100 indicated extreme limitations. The MCID for subjects with multi-directional shoulder instability is 220 (28). The MCID for the NPRS for patients with shoulder pain has been found to be 2.17 (29) with a test-retest reliability of 0.74 (22). While there is some overlap between these outcome measures, each examines different facets of upper extremity function. The UEFI focuses on difficulty performing common activities of daily living (ADLs). The SPADI also focuses on ADLs but includes a section on pain severity during activity. The QuickDASH includes questions specific to work and social activities that is not present in the other outcome measures and that was relevant to our patient population. Finally, the WOSI was the only measure that specifically inquired about instability and the emotional impact of shoulder pain and disability on the individual, which may be profound (30). Using 4 different outcome measures assured that multiple aspects of function would be assessed.

**Figure 1:**
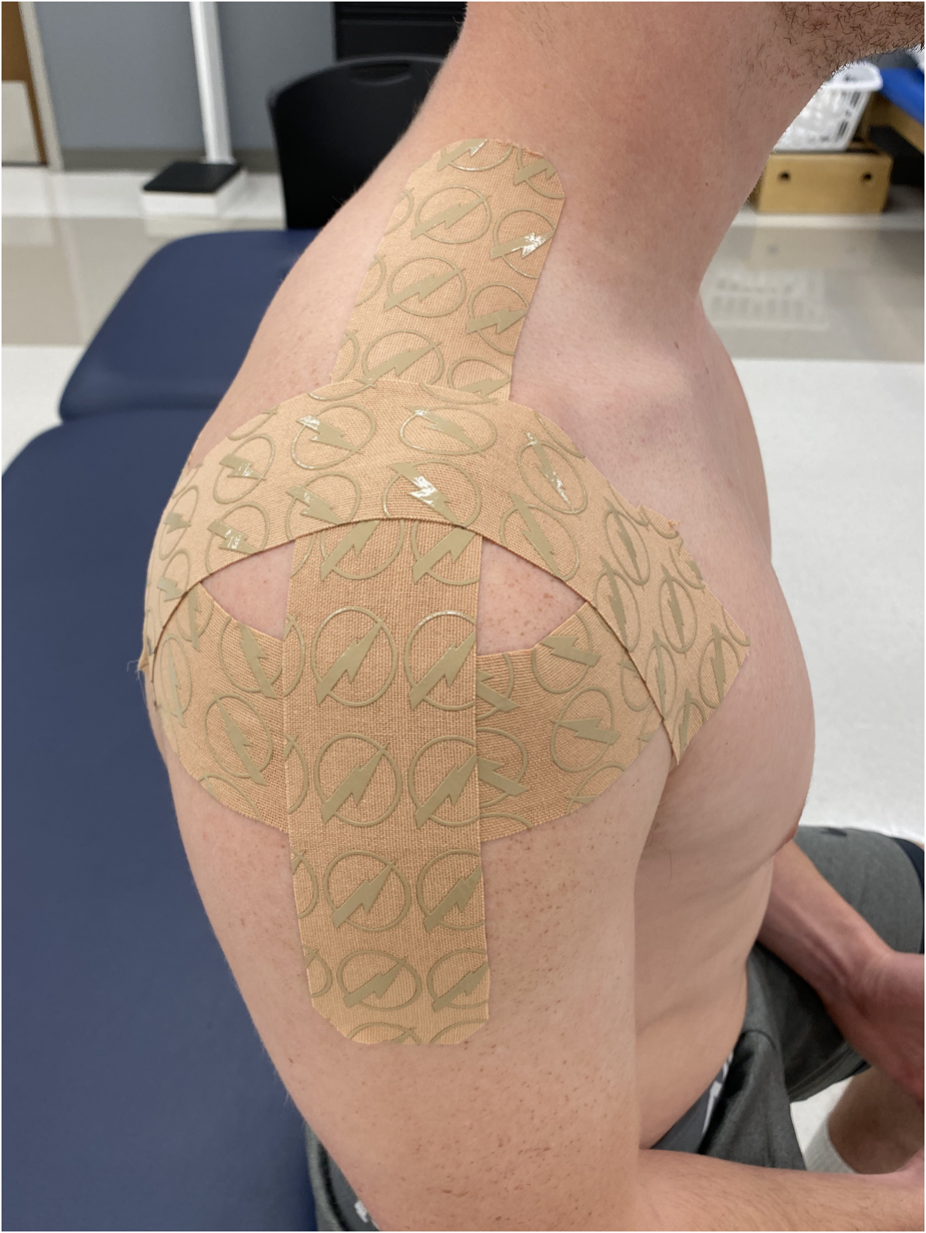
Experimental shoulder taping.

**Figure 2:**
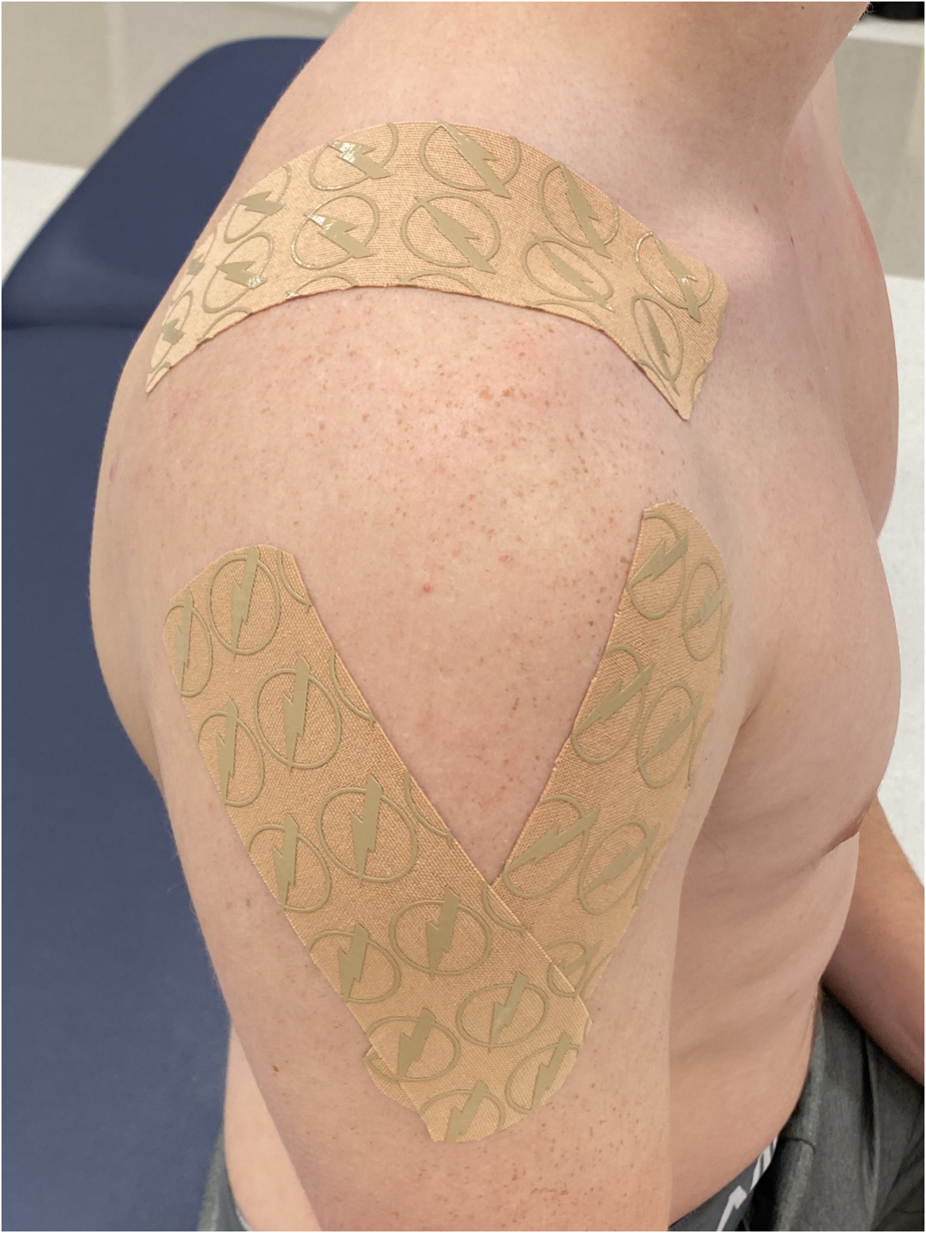
Control shoulder taping.

After the NPRS values were recorded and PRO measures completed, all subjects received either the experimental shoulder KT procedure or the control shoulder taping. All subjects presented with bilateral shoulder pain, therefore, both shoulders of each participant were taped using the same taping protocol and applied by the same therapist. All taping procedures utilized the Thrive kinesiology tape (Melrose, MA) applied with light tension. The experimental taping protocol involved the first strap being applied from the lateral-inferior aspect of the clavicle and wrapping around to the medial aspect of the scapula posteriorly. The second strap was applied on the upper trapezius and extended laterally to the area of the deltoid tuberosity, and the third strap was placed on the anterior aspect of the humeral head and placed superiorly over the acromion to the end on the medial scapula (Figure 1). The control taping involved the first strap being applied from the distal clavicle to the deltoid tuberosity, the second strap from the superior-lateral border of the scapula to the deltoid tuberosity, and the third strap over the upper trapezius from the clavicle to the spine of the scapula (Figure 2). Both tapings used approximately the same amount of tape; the key difference being that the KT did not cross the glenohumeral joint line in the control group.

After receiving the tape application, the current pain level rating was reassessed using the NPRS. The participants were asked to maintain the tape on their shoulders unless it became uncomfortable or caused skin irritation. The participants returned 48 h later to repeat the PRO measures again.

### Statistical approach

Data were analyzed using SPSS (IBM SPSS Statistics for Windows, Version 26.0). Descriptive statistics were performed on all demographic data (height, weight, and gender). Initially, a Shapiro-Wilk test was performed to assess for the normality of the baseline data, with parametric analysis being used for the normally distributed data and non-parametric analysis reserved for the data which were not normally distributed. Baseline characteristics were assessed between the groups using a series of independent sample T-Tests to ensure both groups were equal at baseline. For the UEFI, SPADI, QuickDASH, WOSI, and NPRS for average and worst pain over 24 h, a 2 × 2 (group x time) mixed ANOVA was performed for each dependent variable to compare the effects of the different taping procedures at baseline and 48 h following treatment. For the current pain level, a 2 × 3 (group x time) mixed ANOVA was performed to compare the effects of the different taping procedures at baseline, immediately following, and 48 h post treatment.

## Results

Demographic data and baseline PRO measures are outlined in Table 1. A total of 29 individuals (28 female) with hEDS and bilateral shoulder pain were recruited to participate in this study. After randomization, 15 were allocated to the control taping group with the remaining 14 allocated to the experimental group. Results from the Shapiro-Wilk tests indicated that each variable for each group were normally distributed, allowing for parametric analysis of the data. Results of the independent sample T-tests (Table 1) demonstrated that there were no differences between the groups on any of the anthropometric or baseline outcome measures. There was, however, a significant difference in age between groups.

**Table 1:** Demographic and baseline outcome measure data.

Variable	Group	Result mean (SD)	T (*p*-Value) mean (SD)
Age (yrs)	Experimental	34.71 (10.93)	−2.207 (.036)
	Control	47.33 (18.6)	
	Total	41.24 (16.4)	
Height (cm)	Experimental	159.74 (8.1)	−2.057 (.051)
	Control	165.18 (5.8)	
	Total	162.56 (7.4)	
Weight (kg)	Experimental	66.26 (20.2)	−1.096 (.28)
	Control	74.57 (20.6)	
	Total	70.56 (20.5)	
NPRS Average	Experimental	4.36 (1.87)	0.574 (0.59)
	Control	4.00 (1.65)	
	Total	4.17 (1.73)	
NPRS Worst	Experimental	6.71 (2.30)	−0.287 (0.78)
	Control	6.93 (1.79)	
	Total	6.83 (2.02)	
NPRS Current Pre-Tape	Experimental	3.50 (2.53)	−0.04 (0.97)
	Control	3.53 (1.89)	
	Total	3.52(2.18)	
UEFI	Experimental	44.86 (19.51)	−1.01 (0.32)
	Control	51.33 (14.75)	
	Total	48.21 (17.22)	
SPADI	Experimental	45.82 (23.70)	0.985 (0.33)
	Control	37.54 (21.58)	
	Total	42.54 (22.61)	
QuickDASH	Experimental	51.46 (20.97)	1.07 (0.29)
	Control	43.74 (17.74)	
	Total	47.47 (19.42)	
WOSI	Experimental	1248.64 (404.86)	0.829 (0.41)
	Control	1127.00 (385.18)	
	Total	1185.72 (392.57)	

SD, Standard deviation;Yrs, years; cm, centimeter; kg, kilogram; NPRS, Numeric Pain Rating Scale; UEFI, Upper Extremity Functional Index; SPADI, Shoulder Pain and Disability Index; WOSI, Western Ontario Shoulder Instability Index.

At the follow-up visit, 48 h after the KT application, the tape remained on all subjects. The skin under the tape was observed. A total of 3/58 (5.2%) shoulders had redness, of which 2 of the 3 had small blistering of the skin, which resolved within 2–4 days after tape removal. There were no other adverse effects. Table 2 summarizes the results of the 2 × 2 mixed ANOVA for the PRO measures and average and worst pain levels over the last 24 h. The results of the UEFI demonstrated that while there was a significant main effect for time [*F*_(1,27)_ = 28.896, *p* = <0.001, *ƞ*_p_^2^ = .517], there was no significant interaction effect [*F*_(1,27)_ = 1.164, *p* = .290, *ƞ*_p_^2^ = .041]. This indicated that when the grouping variable was ignored, there were significant improvements in the UEFI but neither group improved more than the other. For the SPADI, while there was also a significant main effect for time [*F*_(1,27)_ = 33.897, *p* = <0.001, *ƞ*_p_^2^ = .557], the interaction effect was not insignificant [*F*_(1,27)_ = .241, *p* = .627, *ƞ*_p_^2^ = .009]. This once again indicates that while both groups improved, neither did so more than the other. Similarly for the QuickDASH and WOSI, the main effect for time was significant ([*F*_(1,27)_ = 31.072, *p* = <0.001, *ƞ*_p_^2^ = .535] and [*F*_(1,27)_ = 69.182, *p* = <0.001, *ƞ*_p_^2^ = .719] respectively), while the interaction effect was not significant ([*F*_(1,27)_ = .123, *p* = .728, *ƞ*_p_^2^ = .005] and [*F*_(1,27)_ = 2.411, *p* = <.132, *ƞ*_p_^2^ = .082] respectively).

**Table 2:** 2 X 2 mixed ANOVA comparing outcome measures pre-taping to 48-hour post-taping.

Variable	Group	Pre-Intervention mean (SD)	Follow-Up mean (SD)	*F* value (Within-Subject)	F value (Interaction)
UEFI	Experimental	44.86 (19.51)	55.08 (20.62)	28.89 (<0.001)	1.164 (0.29)
	Control	51.33 (14.75)	58.13 (14.08)		
SPADI	Experimental	45.82 (23.70)	20.28 (26.21)	33.9 (<0.001)	0.241 (0.63)
	Control	37.54 (21.58)	24.41 (18.46)		
QuickDASH	Experimental	51.46 (20.97)	37.09 (25.52)	31.07 (<0.001)	0.123 (0.73)
	Control	43.74 (17.74)	31.08 (16.14)		
WOSI	Experimental	1,248.64 (404.86)	684.29 (479.84)	69.18 (<0.001)	1.422 (0.13)
	Control	1,127 (385.18)	740.2 (381.99)		
NPRS Average	Experimental	4.36 (1.87)	3.29 (2.09)	9.225 (0.005)	0.064 (0.80)
	Control	4 (1.65)	2.73 (1.79)		
NPRS Worst	Experimental	6.71 (2.30)	4.93 (2.59)	16.66 (<0.001)	0.815 (0.38)
	Control	6.93 (1.79)	4.13 (1.96)		

UEFI, Upper Extremity Functional Index; SPADI, Shoulder Pain and Disability Index; WOSI, Western Ontario Shoulder Instability Index; NPRS, Numeric Pain Rating Scale.

The average NPRS score over the last 24 h demonstrated significant improvements over time [*F*_(1,27)_ = 9.225, *p* = 0.005, *ƞ*_p_^2^ = .225]; however, there was no significant interaction effect meaning both groups improved at the same rate [*F*_(1,27)_ = .064, *p* = .802, *ƞ*_p_^2^ = .002]. Likewise, the results for the worst pain over the last 24 h demonstrated a significant improvement over time [*F*_(1,27)_ = 16.662, *p* = <0.001, *ƞ*_p_^2^ = .382] but no difference between the groups [*F*_(1,27)_ = .815, *p* = .375, *ƞ*_p_^2^ = .029]. For current pain (Table 3), the 2 × 3 Mixed ANOVA demonstrated a significant effect for time [*F*_(2,54)_ = 4.059, *p* = .023, *ƞ*_p_^2^ = .131] with no significant interaction term [*F*_(2,54)_ = .042, *p* = .959, *ƞ*_p_^2^ = .002]. A post-hoc analysis found that there were significant differences between the pre-intervention pain and immediate post-intervention pain rating [mean difference = 0.86 (0.174–1.545), *p* = 0.016], as well as the pre-intervention and 48-hour post-intervention pain rating [mean difference = 0.962 (0.123–1.801), *p* = 0.026]. In contrast, there were no differences between the immediate post-intervention and 48-hour post intervention pain ratings [mean difference = 0.102 (−.648–.853), *p* = 0.782]. This indicated that the pain reduction following taping was immediate, lasted over the 48 h until follow up, but did not improve further. Note that the supplemental analyses for all outcome measures were conducted again, using age as a covariate and the findings did not change. Therefore, we concluded that age, while significantly different between groups, did not affect the results presented.

**Table 3:** Current numeric pain rating scores.

	Group	N	Mean (SD)
NPRS Current Pre-Tape	Experimental	14	3.50 (2.53)
	Control	15	3.53 (1.89)
	Total	29	3.52 (2.18)
NPRS Current Immediately Post-Tape	Experimental	14	2.71(1.44)
	Control	15	2.60 (1.84)
	Total	29	2.66 (1.63)
NPRS Current 48 Hours Post-Tape	Experimental	14	2.64 (2.34)
	Control	15	2.47 (2.10)
	Total	29	2.55 (2.18)

SD, standard deviation; NPRS, Numeric Pain Rating Scale.

## Discussion

The primary purpose of this study was to assess the efficacy and short-term effects of two different KT techniques on shoulder pain and function in individuals with hEDS and shoulder pain. Function was assessed using 4 valid, reliable, and responsive outcome measures that examine different dimensions of upper extremity function. The average baseline WOSI score for our study was 1,185 which correlates closely with other current research studying patients with EDS, adding validity to our results (31). In both the control and experimental group, there was significant improvement in function from pre-intervention to 48 h post tape (*p* < 0.001) with large effect sizes (*ƞ*_p_^2^ = .517–.719) for all outcome measures. The improvements in both taping groups surpassed the MCID for the SPADI, QuickDASH, and WOSI. For the UEFI, both groups showed improvement, however only the experimental group surpassed the MCID. This is of questionable significance as there was no statistical difference between the groups and the improvements produced large effect sizes.

The pain ratings for worst pain, average pain, and current pain all showed significant statistical improvement at each time point with large effect sizes (Tables 2, 3). However, only the worst pain surpassed the MCID. We chose the NPRS MCID based on values obtained from a study of patients with surgical and non-surgical shoulder pain, as this was the target joint for the KT (29). However, the patients in this study could also be appropriately assigned into a chronic pain category where a reduction of 1-point or 15% represents the MCID (32). If 15% were used, the MCID would have been surpassed in all 3 of the pain ratings. One explanation for this apparent discrepancy is that cut-off scores associated with MCID are not uniform and may vary based on the baseline pain levels and chronicity. Studies have shown that patients with higher baseline pain require greater reductions in pain to be considered clinically meaningful compared with patients with lower levels of pain (32, 33). In our study, the baseline pain values for average pain was 4.17/10 and for current pain was 3.52/10, both of which would be considered low or at the beginning of a moderate pain level; whereas the worst pain (6.83/10) was closer to a high pain level.

While the improvements in function and pain seem clear, the mechanism by which KT operates remains unclear. The tape is very elastic, and it is usually placed on the skin with little pressure making a biomechanical improvement in stability unlikely. Another proposed mechanism is through improved proprioception (13, 14). This was the reason why the control tape deliberately did not cross the glenohumeral joint. However, just placing the tape on the skin may have affected sensorimotor integration and impacted proprioception and motor control through cutaneous afferents (34, 35). Previous work has shown that individuals with shoulder instability demonstrate sensorimotor control deficiencies (36, 37) and that taping can help reduce those deficiencies (38). Furthermore, these effects are seen regardless of the direction and tension of the taping applied to the shoulder (39). This is supported by other studies comparing experimental KT tape and placebo tape that have showed improvement in both groups (40, 41). This however may not explain the immediate reduction in pain post-taping. Psychological effects, placebo, and the Hawthorne effect can also not be ruled out in this study (42).

Our experience with subjects with EDS indicates that when concerning the near constant multi-joint pain, any relief is welcome. However, as with any intervention, the reward must outweigh the risk. Skin fragility is a common finding in patients with EDS (43) and 5.2% of the taped shoulders demonstrated adverse skin reactions to the tape adhesive at the 48-hour follow up. The skin reactions in this study were minor and resolved within 4 days after removal. It is important to apply the tape with minimal stretch in this patient population and there may be a difference in response based on the brand of tape used.

For those who opt to use KT, one benefit is that it is relatively inexpensive. The Thrive Kinesiology Tape used in this study costs approximately $30.00 U.S. Dollars per roll and several shoulder applications can be performed with 1 roll of tape (https://thrivetape-eds.com). The subject can also be taught how to independently or with minimal assistance apply the tape, improving self-efficacy. In our study, the control tape was just as effective as the standardized experimental shoulder tape. Combining our results with those of other studies, it appears that the specific taping technique and direction of tape application are of little significance. Furthermore, with no consensus on how much tape tension is most beneficial (44, 45), the patient has freedom to experiment with different configurations and varying amounts of tension that may yield the optimal short-term benefit with little skin irritation.

Our study does have limitations. The shoulder apprehension test applied on day 1 could have increased symptoms which then subsided over the 48-hour test window contributing to the improvement seen. This was minimized by the test being applied carefully; and after testing no participant reported an increase in shoulder pain. While the subjects were blinded to which taping procedure they received, there was no true control group (i.e., a group that did not receive KT) and the follow up was over only 48 h. Additionally, the subjects may have researched different taping techniques for the shoulder to self-determine if they were in the control or experimental group which may have affected their response to the treatment. They were not asked at any point which group they believed they were allocated to. The tape application also could have varied slightly between shoulders and participants; however, this was minimized by having the same researcher who was experienced with KT perform all the taping. The QuickDASH, SPADI, and WOSI questionnaires all ask about function over the preceding week; however, we re-tested the participants after 48-hours and recall bias may have affected the responses. The UEFI specifically asks about symptoms on the day the questionnaire is completed and also showed significant improvement. Finally, the sample size consisted of only 29 individuals. However, all subjects had bilateral shoulder pain, so the KT tape was applied to 58 shoulders. There was a significant difference in age between the experimental and control groups; however, after controlling for age using an ANCOVA, there still remained no difference in outcomes between the groups. Future research could include adding a true control group (no KT) and examining proprioception using standardized tests pre- and post-taping.

## Conclusion

Hypermobile EDS is a common condition manifesting with multi-joint pain, often including the shoulder. KT is an inexpensive and relatively safe intervention that may can be applied by patients independently or with minimal assistance and offer temporary improvements in pain and function.

## Data Availability

The raw data supporting the conclusions of this article will be made available by the authors, without undue reservation.
